# Intraoperative Oxygen Delivery and Acute Kidney Injury after Liver Transplantation

**DOI:** 10.3390/jcm9020564

**Published:** 2020-02-19

**Authors:** Won Ho Kim, Ho-Jin Lee, Hee-Chul Yoon, Kook Hyun Lee, Kyung-Suk Suh

**Affiliations:** 1 Department of Anesthesiology and Pain Medicine, Seoul National University Hospital, Seoul National University College of Medicine, Seoul 03080, Korea; zenerdiode033@gmail.com (H.-J.L.); heechul@snu.ac.kr (H.-C.Y.); leekh@snu.ac.kr (K.H.L.); 2 Department of Surgery, Seoul National University Hospital, Seoul National University College of Medicine, Seoul 03080, Korea; kssuh@snu.ac.kr

**Keywords:** acute kidney injury, liver transplantation, oxygen delivery, oxygen content

## Abstract

Although intraoperative hemodynamic variables were reported to be associated with acute kidney injury (AKI) after liver transplantation, the time-dependent association between intraoperative oxygen delivery and AKI has not yet been evaluated. We reviewed 676 cases of liver transplantation. Oxygen delivery index (DO_2_I) was calculated at least ten times during surgery. AKI was defined according to the Kidney Disease Improving Global Outcomes criteria. The area under the curve (AUC) was calculated as below a DO_2_I of 300 (AUC < 300), 400 and 500 mL/min/m^2^ threshold. Also, the cumulative time below a DO_2_I of 300 (Time < 300), 400, and 500 mL/min/m^2^ were calculated. Multivariable logistic regression analysis was performed to evaluate whether AUC < 300 or time < 300 was independently associated with the risk of AKI. As a sensitivity analysis, propensity score matching analysis was performed between the two intraoperative mean DO_2_I groups using a cutoff of 500 mL/min/m^2^, and the incidence of AKI was compared between the groups. Multivariable analysis showed that AUC < 300 or time < 300 was an independent predictor of AKI (AUC < 300: odds ratio [OR] = 1.10, 95% confidence interval [CI] 1.06–1.13, time < 300: OR = 1.10, 95% CI 1.08–1.14). Propensity score matching yielded 192 pairs of low and high mean DO_2_I groups. The incidence of overall and stage 2 or 3 AKI was significantly higher in the lower DO_2_I group compared to the higher group (overall AKI: lower group, *n* = 64 (33.3%) vs. higher group, *n* = 106 (55.2%), *p* < 0.001). In conclusion, there was a significant time-dependent association between the intraoperative poor oxygen delivery < 300 mL/min/m^2^ and the risk of AKI after liver transplantation. The intraoperative optimization of oxygen delivery may mitigate the risk of AKI.

## 1. Introduction

Acute kidney injury (AKI) is a clinically relevant complication after liver transplantation associated with mortality, poor graft survival [1,2,3,4,5], and chronic kidney disease [6,7]. Although its incidence has been reported to be as high as 68% [1,2,8,9,10,11,12], an effective treatment has not yet been established. Therefore, it is important to identify a modifiable risk factor and to prevent its occurrence.

Intraoperative hemodynamic variables have been investigated as modifiable risk factors in patients undergoing liver transplantation. Intraoperative arterial blood pressure, baseline central venous pressure, baseline right ventricular end-diastolic pressure and mixed venous oxygen saturation (SvO_2_) during the anhepatic phase were reported to be associated with the development of posttransplant AKI [13,14]. Preoperative anemia and intraoperative transfusion amounts have also been reported as risk factors of AKI [9,13]. All these hemodynamic variables are related to cardiac output and arterial oxygen content, which are components of intraoperative oxygen delivery [15]. Poor oxygen delivery to the kidneys is one of the potential mechanisms of the development of AKI. Albeit in cardiac surgeries with cardiopulmonary bypass, a previous study reported the association between time-dose response of oxygen delivery and AKI after cardiac surgery [16]. The area under the curve below the threshold of an oxygen delivery index (DO_2_I) of 300 mL/min/m^2^ was significantly different between those with and without AKI. A following randomized trial for the goal-directed perfusion of a cardiopulmonary bypass aimed at maintaining DO_2_I at ≥280 mL/min/m^2^ reduced the incidence of AKI [17]. However, these hypotheses have not been evaluated in patients undergoing liver transplantation.

During liver transplantation, frequent changes in cardiac output and hemoglobin concentration occur due to the clamping of the inferior vena cava and significant surgical bleeding [18]. These fluctuations may result in poor oxygen delivery to the kidney, which is vulnerable to ischemic injury. Therefore, poor intraoperative oxygen delivery could be a strong risk factor of posttransplant AKI. The adequacy of major organ oxygenation could be estimated by DO_2_I adapted to oxygen demand [19]. However, the strategy for optimizing DO_2_I during liver transplantation surgery adapted to meet oxygen needs is still unknown [20]. The prognostic implication of intraoperative DO_2_I on postoperative AKI and mortality as well as the optimal target of intraoperative DO_2_I during liver transplantation has not yet been investigated.

Therefore, in this retrospective observational study, we attempted to evaluate the association between intraoperative DO_2_I and the risk of AKI after liver transplantation and find the optimal cutoff of DO_2_I which is associated with the risk of AKI. To evaluate this time-dependent association, the area under the curve below a threshold of DO_2_I, and the cumulative time below a threshold of DO_2_I were considered as potential predictors of DO_2_I.

However, the etiology of AKI after liver transplantation is known to be multifactorial [21], and the baseline graft qualities, including the type of donor graft (deceased vs. living donor; circulatory vs. brain death) [12] and warm and cold graft ischemic time [9,14,22,23], are associated with AKI. Therefore, we sought to adjust perioperative variables by performing propensity score matching analysis.

## 2. Materials and Methods

### 2.1. Study Design

The institutional review board of Seoul National University Hospital approved our retrospective observational study (1904-073-1026). We retrospectively reviewed the electronic medical records of 1041 consecutive patients who underwent living or deceased donor liver transplantation at our institution between 2007 and 2015. The need for informed consent was waived. Among the 1041 patients, those with preoperative renal dysfunction, including acute kidney injury, hepatorenal syndrome or a need for hemodialysis (*n* = 105), were excluded. Also, the patients for whom a pulmonary artery catheter was not inserted during surgery (*n* = 155) were excluded. The patients with cardiac output recorded less than ten times were excluded (*n* = 105). The remaining 676 cases with living (*n* = 481, 71.2%) and deceased donors (*n* = 195, 28.8%) were included in our analysis.

### 2.2. Anesthesia and Surgical Technique

Anesthesia was induced and maintained with propofol or an inhalational agent. Rocuronium was used to maintain the neuromuscular blockade. Volume-controlled ventilation was maintained with a tidal volume of 6–8 mL/kg. Arterial-line catheters were inserted into both radial and femoral arteries. A pulmonary artery catheter was inserted routinely, placed in the right internal jugular vein. The continuous cardiac index and right ventricle-associated variables were monitored using the Vigilance II monitor (Edward Lifesciences, Irvine, CA, USA). The continuous infusion of dopamine or epinephrine or norepinephrine was used to treat hypotension according to the monitored cardiac index, SvO_2_ and systemic vascular resistance (SVR). During the study period, the threshold for intraoperative red cell transfusion was consistent at 20%.

A histidine–tryptophan–ketoglutarate solution was used for the donor liver graft. The piggyback technique was used to anastomose the donor and graft vessels. The end-to-end anastomosis of the hepatic artery and duct-to-duct anastomosis of the bile duct were performed in succession. During surgery, 20 mg of basiliximab (Simulect, Novartis Pharma B.V., Arnhem, The Netherlands) and 500 mg of methylprednisolone (Solumedrol, Pfizer, Ballerup, Denmark) were used for the induction of immunosuppression. We initiated postoperative immunosuppression with calcineurin inhibitor of tacrolimus with mycophenolate mofetil on the first postoperative day.

### 2.3. Data Collection and Study Outcomes

According to the previous literature, data related to demographic or perioperative variables known to be associated with postoperative renal dysfunction were collected [1,2,9,11,21,24,25]. Preoperatively, the Model for End-stage Liver Disease (MELD) score, the Child–Turcotte–Pugh (CTP) score, and the CTP classification were collected for all patients [26]. History of hypertension, diabetes mellitus, preoperative serum albumin, graft macrosteatosis, warm ischemic time, cold ischemic time, graft-to-recipient body weight ratio (GRWR), intraoperative blood loss, the amount of intraoperative transfusion, crystalloid and colloid administration were investigated.

The primary outcome variable was postoperative AKI, defined according to the Kidney Disease Improving Global Outcomes criteria, which have been validated in liver transplantation [7,8]. We defined postoperative AKI based on the postoperative increase in serum creatinine (Stage 1: 1.5–1.9; stage 2: 2–2.9; stage 3: more than 3-fold increase on baseline, respectively) within the first 7 days after transplantation. The most recent preoperative serum creatinine measured was used as a baseline. Other postoperative clinical outcome variables included the length of the intensive care unit (ICU) stay, the length of hospital stay, early allograft dysfunction [27], and in-hospital all-cause mortality. The incidence of chronic hemodialysis and new-onset chronic kidney disease during one year after transplantation were also compared between groups. Chronic kidney disease was defined as a decrease in eGFR < 60 mL/min/1.73 m^2^ or the initiation of chronic hemodialysis [28]. The decrease in eGFR should be identified by at least two consecutive measurements separated by an interval of at least three months [29].

Oxygen delivery was calculated according to the following equation using cardiac output values at ten time points during surgery as follows: anesthesia induction (T1), 1 h after anesthesia induction (T2), 30 min (T3) and 1 h (T4) after the beginning of the anhepatic phase, 5 min before (T5) and after (T6) graft reperfusion, 20 min after reperfusion (T7), 40 min after reperfusion (T8), 5 min after the completion of biliary reconstruction(T9), and at the end of surgery (T10). Cardiac output was measured by the thermodilution technique. The results of the most recent arterial blood gas analysis were used for the partial pressure of arterial oxygen (PaO_2_) and arterial oxygen saturation (SaO_2_, %). The hemoglobin level nearest to the time point of measurement was used for this calculation [30].

$$\mathrm{Oxygen}\;\mathrm{delivery}\;\mathrm{index}\;\left(\mathrm{mL}/\min/m^{2}\right)=\left(1.39\times \mathrm{Hemoglobin}\times \mathrm{Sa}O_{2}+0.0031\times \mathrm{Pa}O_{2}\right)\times \mathrm{Cardiac}\;\mathrm{index}\;\times 10$$

### 2.4. Statistical Analysis

SPSS software version 25.0 (IBM Corp., Armonk, NY, USA), STATA/MP version 15.1 (StataCorp, College Station, TX, USA) and Medcalc Statistical Software version 18.6 (MedCalc Software bvba, Ostend, Belgium) were used to analyze the data. For all analyses, *p* < 0.05 was considered statistically significant. The Shapiro–Wilk test was used to determine the normality of the data. Fisher’s exact test or χ^2^ test was used to compare the incidence variables shown in Table 1. Comparisons of the continuous variables shown in Table 1 were performed using the Mann–Whitney *U* test. Missing data were present in <5% of records. Missing values of continuous variables were replaced by the sex-and age-specific median values, and incidence data were assigned the modes of sex- and age-specific values.

Firstly, time-dependent changes in the values of DO_2_I, cardiac index and arterial oxygen content at ten time points were depicted between those with and without AKI. As a component of arterial oxygen content, time-dependent changes in hemoglobin, PaO_2_, and SaO_2_ were also compared between those with and without AKI. Values at each time point were compared using the Mann–Whitney *U* test and multiple comparisons were adjusted by Bonferroni correction. For each variable, *p* < 0.005 was considered to be significant.

Secondly, time-dependent changes in the intraoperative DO_2_I were summarized in terms of the following variables: mean, nadir, standard deviation, area under the curve (AUC) below the threshold of DO_2_I of 500 (AUC < 500), 400 (AUC < 400) and 300 (AUC < 300) mL/min/m^2^, and cumulative time below the threshold of DO_2_I of 500 (time < 500), 400 (time < 400) and 300 (time < 300) mL/min/m^2^ [31]. The AUC was calculated as described previously [32]. The mean DO_2_I was calculated as the simple average of the observed values. The nadir DO_2_I during surgery was determined as the lowest point during measurement. To find the relevant DO_2_I-related variable and its cutoff, AUCs of these variables with varying cutoffs were compared to each other regarding the area under the receiver operating characteristic curve and the variable with the largest area under the receiver operating characteristic curve was determined to be entered into the multivariable analysis. The candidate thresholds of AUC < 300, 400 and 500 or time < 300, 400, 500 were determined according to the cutoff of normal reference value and previous studies [16,33]. The thresholds of 400 and 500 were determined as the close values near the 10th and 25th percentile of our distribution of mean DO_2_I.

Thirdly, multivariable binary logistic regression analysis was performed to evaluate whether intraoperative DO_2_I-related parameters are independent predictors of AKI. All of the variables in Table 1, as well as the intraoperative hemodynamic variables previously reported to be associated with AKI, were considered in the analysis [13]. No univariable screening or variable selection process was performed. The calibration of the prediction model was assessed using Hosmer–Lemeshow goodness-of-fit statistics. The performance of the model was assessed with Nagelkerke′s R^2^.

Fourthly, as a sensitivity analysis to evaluate the association between DO_2_I and AKI, we performed propensity score matching between two intraoperative mean DO_2_I groups, using a cutoff of 500 mL/min/m^2^. The following covariates were considered for matching: recipient age, gender, body mass index, alcoholic cirrhosis, hepatocellular carcinoma, hypertension, diabetes mellitus, hemoglobin, serum albumin level, MELD score, child class, preoperative beta-blocker, diuretics, living/deceased donor, previous abdominal surgery, estimated GRWR, operation time, cold/warm ischemic time, dose of epinephrine, and the level of red blood cell transfusion. Matching was performed by using the nearest neighbor algorithm with a caliper width equal to 0.2 of the pooled standard deviation of the logit of the propensity score. The incidence of AKI and other clinical outcomes including the length of hospital and ICU stay, early allograft dysfunction and in-hospital mortality were compared between the matched groups.

Finally, cubic spline function curves were drawn to evaluate the adjusted relationship between DO_2_I-related parameters including AUC < 300, time < 300, mean and nadir DO_2_I during surgery as continuous variables and the probability of development of AKI. The covariates used for logistic regression analysis were considered.

## 3. Results

**Figure 1:**
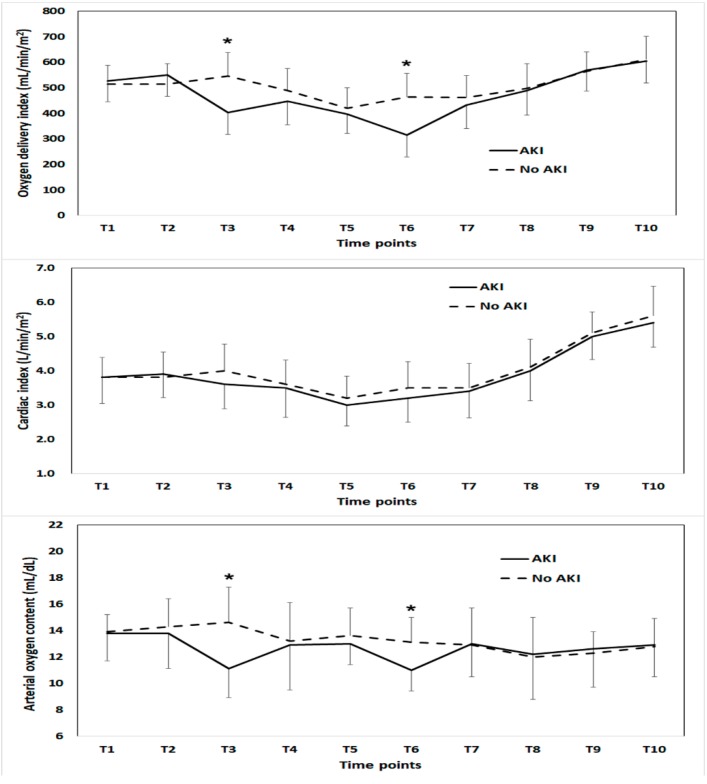
Comparison of oxygen delivery index (upper), cardiac index (middle) and arterial oxygen content (lower) during liver transplantation between the patients with and without acute kidney injury. The time points compared were as follows: anesthesia induction (T1), 1 h after anesthesia induction (T2), 30 min (T3) and 1 h (T4) after the beginning of the anhepatic phase, 5 min before (T5) and after (T6) graft reperfusion, 20 min after reperfusion (T7), 40 min after reperfusion (T8), 5 min after the completion of biliary reconstruction(T9), and at the end of surgery (T10). * Significant difference between groups.

Among 676 patients in this study, 275 patients (40.7%) were diagnosed with AKI; stage 1 (*n* = 207, 30.6%), stage 2 (*n* = 48, 7.1%), and stage 3 (*n* = 20, 3.0%). The patients’ baseline clinical characteristics and perioperative parameters were compared according to the intraoperative mean DO_2_I groups in Table 1. Figure 1 shows the time-dependent change in DO_2_I, cardiac index and arterial oxygen content. Supplemental Figure S1 shows the time-dependent change in hemoglobin, PaO_2_ and SaO_2_. There were significant differences in DO_2_I and arterial oxygen content during the anhepatic phase (T3) and immediately after reperfusion (T6).

Supplemental Figures S2 and S3 show the comparison of areas under the receiver operating characteristic curve of the AUC < 300, 400, 500, time < 300, 400, 500, mean, nadir, and standard deviation of DO_2_I. AUC < 300 and time < 300 showed the largest area under the receiver operating characteristic curve and were entered into the multivariable logistic regression model.

Table 2 shows the results of multivariable logistic regression analysis for AKI after liver transplantation in all patients. AUC < 300 was identified as an independent predictor of postoperative AKI (odds ratio [OR] = 1.10, 95% confidence interval [CI] 1.06–1.13). When time < 300 was considered in the model instead of AUC < 300, it was also significant (OR 1.10, 95% CI 1.08–1.14). Our regression model with AUC < 300 showed good performance (Nagelkerke′s R^2^ = 0.286) and calibration (Hosmer–Lemeshow goodness-of-fit: χ^2^ = 13.4, *p* = 0.097).

Patients with a low mean DO_2_I during surgery were associated with a significantly higher incidence of AKI and required longer periods of hospital and ICU stay (Table 3). After propensity score matching, 192 pairs between low and high mean DO_2_I groups remained (Supplemental Table S1). Histograms of the distribution of standardized differences and covariate balance plot before and after matching are presented in Supplemental Figure S4. After matching, there was no unbalanced contributor to propensity scores, with a standardized mean difference of ≥0.20 between groups. In the matched cohort, the incidence of overall and stage 2 or 3 AKI was significantly higher in the lower DO_2_I group compared to higher group (overall AKI: lower group, *n* = 64 (33.3%) vs. higher group, *n* = 106 (55.2%), *p* < 0.001) (Table 2). There were also significant differences in secondary clinical outcomes.

**Figure 2:**
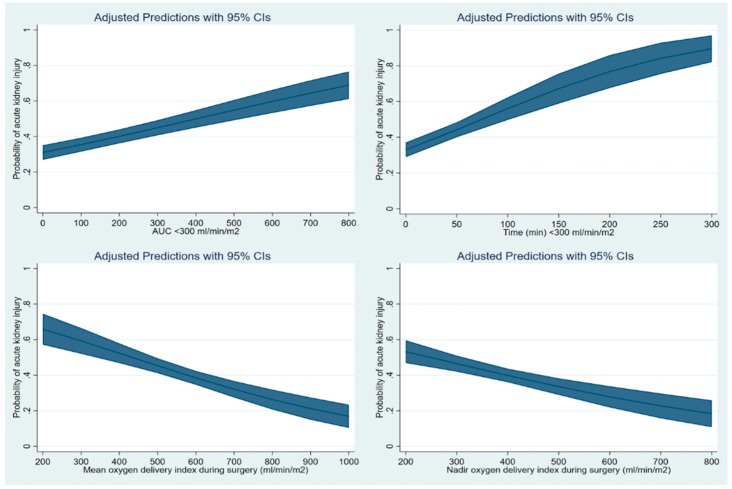
The cubic spline function curves of the multivariable-adjusted relationship between the area under the threshold of oxygen delivery index (DO_2_I) of 300 mL/min/m^2^ (AUC < 300) (upper left) or the cumulative time below the threshold of DO_2_I of 300 mL/min/m^2^ (time < 300) (upper right) or mean DO_2_I during surgery (lower left), or nadir DO_2_I during surgery (lower right) as continuous variables and the risk of acute kidney injury after liver transplantation.

The cubic spine function curve shows that the AUC < 300 or time < 300 showed a positive slope with a linear relationship with the risk of AKI after liver transplantation, while mean and nadir DO_2_I showed a negative slope with the risk of AKI (Figure 2).

The values are expressed as the median [interquartile range] or number (%). The incidence of chronic hemodialysis and chronic kidney disease were determined during one year after transplantation. Chronic kidney disease was defined as a decrease in eGFR < 60 mL/min/1.73 m^2^ or the initiation of chronic hemodialysis [28]. The decrease in eGFR should be identified by at least two consecutive measurements separated by an interval of at least three months [29].

## 4. Discussion

The main finding of our study was that intraoperative DO_2_I-related parameters were associated with AKI as well as other clinical outcomes after liver transplantation, and the cutoff of time-dependent DO2I which was associated with a risk of AKI was 300 mL/min/m^2^. Our finding is clinically relevant because DO_2_I could be a potentially modifiable risk factor of AKI. We demonstrated this association with time-dependent parameters of DO_2_I including the area under the threshold and the cumulative time below the threshold. A continuous linear relationship was found between DO_2_I-related parameters and AKI. This association was also consistent after propensity score matching with baseline perioperative variables. Therefore, intraoperative oxygen delivery could be a modifiable hemodynamic goal to prevent AKI after liver transplantation and could also provide the prognostic values in patients undergoing liver transplantation.

The etiology of postoperative AKI after liver transplantation is known to be multifactorial, including inflammatory response, ischemia-reperfusion injury and nephrotoxic agents [34,35]. Nonetheless, poor oxygen delivery to major organs including the kidneys and a mismatch in oxygen delivery and consumption could be a modifiable mechanism of AKI after liver transplantation. The kidneys are vulnerable to acute ischemic injury due to their low oxygen extraction ratio compared to a high oxygen demand [36]. The kidney lacks the response of increasing perfusion and decreasing oxygen consumption to limited oxygen availability [37].

The hemodynamic management strategy aimed at optimizing renal oxygen delivery could be an option to prevent AKI after liver transplantation. Oxygen delivery is determined by arterial oxygen content and cardiac output, which is comprised of hematocrit concentration, PaO_2_, heart rate and stroke volume. Although the target of maintenance would be slightly different depending on patients’ baseline medical conditions, goal-directed management could be performed to optimize goals. Nonetheless, oxygen delivery is not generally monitored in daily clinical practice. Although all components of oxygen delivery are well monitored, the oxygen delivery may go astray because the true and optimal value of real-time oxygen delivery is not monitored and is thus unknown, and each component of oxygen delivery is monitored at different time points and different intervals. About 34% of our patients had a mean DO_2_I of less than 500, suggesting that the routine anesthetic management of our institutions may have resulted in insufficient oxygen delivery. Our study results demonstrate the independent and robust association between DO_2_I-related variables of time < 300 or AUC < 300 and AKI, as well as other clinical outcomes, suggesting the necessity of monitoring intraoperative DO_2_I during liver transplantation.

It can be argued that maintaining normal hemoglobin and cardiac output would be sufficient to obtain adequate oxygen delivery and monitoring DO_2_I-related parameters may have no further benefit. Although arterial oxygen content and cardiac index are major components of oxygen delivery, we monitored hemoglobin and the cardiac index value at different times, and prediction with time < 300 showed significantly better discrimination and risk classification than prediction with mean hemoglobin and cardiac index. This means that monitoring DO_2_I could be superior to monitoring the hemoglobin and cardiac index. According to the time-dependent comparison of the components of DO_2_I between those with and without AKI, differences in DO_2_I were mainly, but not all, due to significant differences in arterial oxygen content during the anhepatic phase and immediately after reperfusion. The difference in arterial oxygen content was due to differences in hemoglobin concentration and PaO2 and SaO_2_ concentration, although these components were not significantly different between the DO_2_I groups.

Our observations are largely consistent with recent studies investigating the association between intraoperative oxygen delivery and AKI. A retrospective study of cardiac surgery suggested that the time-dose response of DO_2_I is a significant predictor of AKI [16]. A following randomized trial reported that a goal-directed perfusion strategy aimed at maintaining DO_2_I at ≥ 280 mL/min/m^2^ during cardiopulmonary bypass was effective in preventing postoperative AKI [17]. During liver transplantation, previous studies also reported the influence of oxygen delivery on AKI [38,39]. Arterial oxygen content at 5 min after graft reperfusion was independently associated with AKI [38]. However, unlike our study, this study only calculated the arterial oxygen content at five time points during surgery and did not take into account the cardiac output and time-dependent oxygen delivery. Another prospective study also suggested that impaired renal oxygenation immediately after liver transplantation is associated with AKI [39]. The authors introduced a catheter into the renal vein during the immediate postoperative period and found a mismatch between increased renal oxygen consumption and oxygen delivery.

Other studies also indicated the role of impaired renal oxygen delivery in the development of AKI after cardiac surgery or in critically ill patients [33,40,41]. A recent prospective study reported the association between the intraoperative urinary oxygen tension and AKI after cardiac surgery [40]. A longer period of low urinary oxygen tension was observed in patients with AKI. In a recent animal study [41], cardiopulmonary bypass significantly reduced renal blood flow and medullary oxygen tension, although the mean arterial pressure was maintained above 70 mmHg. Thus, it would be more important to maintain oxygen delivery to meet increased renal oxygen consumption, rather than simply maintaining the arterial pressure. In critically ill patients, a higher systemic oxygen delivery in the initial 12 h since the onset of AKI stage 1 was associated with a lower risk of progression to AKI stage 3 [33]. However, after 12 h from the onset of AKI, this association is not significant. Therefore, maintaining sufficient oxygen supply during surgery may be an effective way to reduce AKI before AKI is established and progressed to a higher stage.

The threshold of normal DO_2_I was determined as 300, 400, and 500 in our analysis, considering the normal reference value and previous studies [16,33]. We determined the optimal cutoff of AUC or cumulative time below the DO_2_I by the largest area under the receiver operating characteristic curve. However, 300 mL/min/m^2^ may not be the optimal hemodynamic goal above which intraoperative DO_2_I should be maintained. Under the hyperdynamic systemic circulation in patients with liver cirrhosis [42], sufficient DO_2_I to prevent AKI may differ. The renal oxygen consumption may be increased due to this specific hyperdynamic circulatory environment and fluctuation in preload and cardiac output during surgery [39]. In a healthy population, a certain threshold of the oxygen delivery value exists, after which the oxygen consumption reaches a plateau on the dose-response curve of oxygen delivery and oxygen consumption. This threshold is often considered to be around 250–280 mL/min/m^2^, but it is increased up to 600 mL/min in critically ill patients [16,17,43,44]. Therefore, the optimal threshold should be further ascertained in future studies considering the balance in oxygen delivery and consumption of the kidney during transplantation.

The effect size of AUC < 300 was 1.10 in terms of multivariable adjusted odds ratio and the area under the receiver operating characteristic curve of the AUC < 300 was 0.72. These values are rather small and the optimization of DO_2_I could be less effective than expected. However, the incidence of chronic hemodialysis and new-onset chronic kidney disease after transplantation was also significantly higher in the low DO_2_I group in the matched cohort, suggesting the clinical importance of DO_2_I.

Baseline graft parameters, including the incidence of a living/deceased donor and cold ischemic time, were significantly different between those with high and low mean DO_2_I groups. A deceased donor and cold ischemic time are reported to be associated with an increased risk of AKI [9,12,14,23]. A warm ischemic time of graft from donation after circulatory death is also associated with the severity of postoperative AKI [22]. Although there was no significant difference in the incidence of deceased donor and cold and warm ischemic time in our matched cohort, the effect of baseline graft status should be considered to interpret our results.

Our study results should be interpreted under the following limitations. Firstly, our retrospective observation could not corroborate a causal relationship between the systemic DO_2_I and postoperative AKI. We only provided a hypothesis that the careful optimization of oxygen delivery might reduce the risk of AKI. Prospective randomized trials are needed to test our hypothesis. Secondly, we calculated systemic DO_2_I rather than measuring renal oxygenation at the renal vein or artery. Renal oxygen delivery and consumption may be different from the systemic values. Third, we used serum creatinine to define AKI. Patients with hepatic failure usually have low levels of serum creatinine because of a decreased skeletal muscle mass, creatinine production and lower conversion of creatine to creatinine [45]. In addition, massive fluid administration and transfusions during operation may result in hemodilution of serum creatinine. Fourthly, our patients included a heterogeneous group of living and deceased donors with varying degrees of liver cirrhosis. The association between oxygen delivery and AKI may be different depending on the severity of liver cirrhosis. We compared the incidence of AKI according to time < 300 in the two risk factor groups of MELD score or Child classification and found that there was a significant interaction between these two risk factors (Supplemental Table S2).

## 5. Conclusions

We found a significant and robust time-dependent association between the intraoperative poor oxygen delivery and the risk of AKI after liver transplantation. The optimal cutoff of AUC under DO_2_I or the cumulative time under DO_2_I, determined by the largest area under the receiver operating characteristic curve, was 300 mL/min/m^2^. This association was also significant after propensity score matching. The intraoperative optimization of oxygen delivery may mitigate the risk of AKI after liver transplantation.

## Figures and Tables

**Table 1:** Patient characteristics and perioperative parameters of the patients with a high and low mean oxygen delivery index (mean DO_2_I) during liver transplantation.

Characteristic	Mean DO_2_I ≥ 500 mL/min/m^2^	Mean DO_2_I < 500 mL/min/m^2^	*p*-Value
Sample size	444 (65.7)	232 (34.3)	
Demographic data			
Age, years	53 (47–58)	58 (52–63)	<0.001
Female, *n*	107 (24.1)	81 (34.9)	0.003
Body-mass index, kg/m^2^	23.2 (21.2–25.3)	23.0 (21.0–25.6)	0.711
Etiology of liver disease			
Alcoholic liver cirrhosis, *n*	58 (13.1)	28 (12.1)	0.713
Hepatitis B viral hepatitis, *n*	213 (48.0)	95 (40.9)	0.082
Hepatitis C viral hepatitis, *n*	42 (9.5)	30 (12.9)	0.165
Hepatocellular carcinoma, *n*	217 (48.9)	87 (37.5)	0.005
Cholestatic disease, *n*	11 (2.5)	7 (3.0)	0.679
Non-alcoholic steatohepatitis, *n*	23 (5.2)	8 (3.4)	0.340
Baseline medical status			
Hypertension, *n*	46 (10.4)	49 (21.1)	0.001
Diabetes mellitus, *n*	66 (14.9)	44 (19.0)	0.170
Preoperative hemoglobin, g/dL	11.4 (9.7–13.1)	10.0 (8.7–11.7)	<0.001
Preoperative serum albumin level, mg/dL	3.0 (2.6–3.6)	2.9 (2.5–3.3)	0.001
Model for end-stage liver disease score	13 (9–20)	17 (11–26)	<0.001
Preoperative serum creatinine, mg/dL	0.84 (0.70–1.00)	0.90 (0.74–1.30)	<0.001
Preoperative prothrombin time, INR	1.5 (1.2–1.9)	1.6 (1.3–2.2)	0.016
Preoperative total bilirubin, mg/dL	3.0 (1.6–7.4)	3.5 (1.8–11.2)	0.061
Child-Turcotte-Pugh score	8 (6–10)	8 (7–10)	0.005
Child class, *n* (A/B/C)	153 (34.5)/180 (40.5)/111 (25.0)	54 (23.3)/102 (44.0)/76 (32.8)	0.002
Preoperative LVEF, %	65 (62–68)	65 (63–69)	0.596
Preoperative beta-blocker, *n*	17 (3.8)	27 (11.6)	<0.001
Preoperative diuretics, *n*	12 (2.7)	18 (7.8)	0.005
Previous abdominal surgery, *n*	8 (1.8)	9 (3.9)	0.122
Donor/graft factors			
Living/deceased donor, *n*	352 (79.3)/92 (20.7)	129 (55.6)/103 (44.4)	<0.001
Age, years	30 (22–40)	31 (25–41)	0.118
Estimated GRWR	1.20 (1.03–1.43)	1.22 (1.11–1.48)	0.035
Operation and anesthesia details			
Operation time, h	6.8 (5.9–7.8)	6.2 (5.3–7.7)	0.003
Cold ischemic time, min	77 (65–118)	118 (72–240)	<0.001
Warm ischemic time, min	30 (27–38)	30 (27–33)	0.048
Intraoperative dose of epinephrine bolus, mcg	0 (0–20)	10 (0–40)	<0.001
Intraoperative mean blood glucose, mg/dL	165 (145–180)	156 (140–179)	0.014
Crystalloid administration, mL	3700 (2500–5000)	3700 (2500–6000)	0.096
Colloid administration, mL	0 (0–900)	0 (0–600)	0.526
Bleeding and transfusion amount			
pRBC transfusion, units	5 (0–10)	7 (3–14)	<0.001
FFP transfusion, units	4 (0–10)	5 (0–14)	0.030

The values are expressed as the median [interquartile range] or number (%). INR = international normalized ratio, LVEF = left ventricular ejection fraction, GRWR = graft recipient body-weight ratio, pRBC = packed red blood cells, FFP = fresh frozen plasma.

**Table 2:** Multivariable logistic regression analysis for acute kidney injury after liver transplantation in all patients (*n* = 676).

Variable	Multivariable-Adjusted OR	95% CI	*p*-Value
Body-mass index, kg/m^2^	1.23	1.06–1.20	<0.001
Child class B or C vs. A	1.99	1.16–3.40	0.012
Baseline right ventricular end-diastolic volume, mL	1.02	1.01–1.02	<0.001
Area under curve below DO_2_I of 300 mL/min/m^2^	1.10	1.06–1.13	<0.001
Or cumulative time (per 10 min) below DO_2_I of 300 mL/min/m^2^	1.10	1.08–1.14	<0.001

CI = confidence interval, DO_2_I = Oxygen delivery index, OR = odds ratio. All variables in Table 1 were considered as covariates in the analysis. Area under curve below DO_2_I of 300 mL/min/m^2^ or cumulative time (min) below DO_2_I of 300 mL/min/m^2^ was entered into the analysis alternatively.

**Table 3:** Comparison of postoperative clinical outcomes between the patients with high and low mean intraoperative oxygen delivery index (mean DO_2_I).

	Before Matching			After Matching		
Characteristic	Mean DO_2_I ≥ 500 mL/min/m^2^ (*n* = 444)	Mean DO_2_I < 500 mL/min/m^2^ (*n* = 232)	*p*-Value	Mean DO_2_I ≥ 500 mL/min/m^2^ (*n* = 192)	Mean DO_2_I < 500 mL/min/m^2^ (*n* = 192)	*p*-Value
Length of hospital stay, days	16 (14–24)	21 (16–31)	<0.001	17 (14–26)	21 (16–31)	0.008
Length of ICU stay, days	5 (4–6)	5 (4–8)	<0.001	5 (4–7)	5 (4–8)	<0.001
Acute kidney injury, *n*	142 (32.0)	133 (57.3)	<0.001	64 (33.3)	106 (55.2)	<0.001
Stage 1, *n*	117 (26.4)	90 (38.8)	0.001	52 (27.1)	76 (39.6)	0.009
Stage 2 or 3, *n*	25 (5.6)	43 (18.5)	<0.001	12 (6.3)	30 (15.6)	0.003
Early allograft dysfunction, *n*	6 (1.4)	7 (3.6)	0.074	3 (1.6)	7 (4.3)	0.196
In-hospital mortality, *n*	12 (2.7)	12 (5.2)	0.125	4 (2.1)	10 (5.2)	0.172
Chronic hemodialysis, *n*	5 (1.1)	10 (4.3)	0.012	2 (1.0)	9 (4.7)	0.032
Chronic kidney disease, *n*	99 (22.3)	61 (26.3)	0.005	38 (19.8)	55 (28.6)	0.043

## Supplementary Materials

The following are available online at https://www.mdpi.com/2077-0383/9/2/564/s1, Supplemental Table S1: Patient characteristics and perioperative parameters of the patients with high and low mean oxygen delivery index (mean DO2I) after propensity score matching; Supplemental Table S2: The incidence of acute kidney injury (AKI) according to the groups of high and low model for end-stage liver disease (MELD) score or Child class and cumulative time below intraoperative oxygen delivery index < 300 mL/min/m^2^ (time < 300); Supplemental Figure S1: Comparison of serum hemoglobin concentration (upper), arterial oxygen partial pressure (middle) and arterial oxygen saturation (lower) during liver transplantation between the patients with and without acute kidney injury; Supplemental Figure S2: The receiver operating characteristic curves to predict posttransplant acute kidney injury of the area under the threshold of oxygen delivery index (DO2I) of 500, 400, 300 mL/min/m*2* (AUC < 300, < 400, < 500), and cumulative time (min) below the threshold of DO2I of 500, 400, 300 mL/min/m*2* (time < 300, < 400, < 500); Supplemental Figure S3: The receiver operating characteristic curves to predict posttransplant acute kidney injury of the area under the threshold of oxygen delivery index (DO2I) of 300 mL/min/m*2* (AUC < 300), cumulative time below the threshold of DO2I of 300 mL/min/m*2* (time < 300 min), mean, nadir, and standard deviation (SD) of intraoperative DO2I; Supplemental Figure S4: Histograms of the distribution of standardized differences and covariate balance plot before and after matching.
