# Supplementary material for: Intraoperative Oxygen Delivery and Acute Kidney Injury after Liver Transplantation
Source: J Clin Med. 2020 Feb 19;9(2):564. doi: 10.3390/jcm9020564 (PMC7073538; doi:10.3390/jcm9020564)

Intraoperative oxygen delivery and acute kidney injury after liver transplantation

: Supplemental Materials

Won Ho Kim, [1], **Ho-jin Lee** [1], **Hee-Chul Yoon**, [1], **Kook Hyun Lee**, [1], **Kyung-Suk Suh**, [2]

List	Title	Page
**Supplemental Table S1**	Patient characteristics and perioperative parameters of the patients with high and low mean oxygen delivery index (mean DO_2I) after propensity score matching.	2
**Supplemental Table S2**	The incidence of acute kidney injury (AKI) according to the groups of high and low model for end-stage liver disease (MELD) score or Child class and cumulative time below intraoperative oxygen delivery index < 300 ml/min/m^2 (time <300).	3
**Supplemental Figure S1**	Comparison of serum hemoglobin concentration (upper), arterial oxygen partial pressure (middle) and arterial oxygen saturation (lower) during liver transplantation between the patients with and without acute kidney injury.	4
**Supplemental Figure S2**	The receiver operating characteristic curves to predict posttransplant acute kidney injury of the area under the threshold of oxygen delivery index (DO_2I) of 500, 400, 300 ml/min/m^2 (AUC <300, <400, <500), and cumulative time (minutes) below the threshold of DO_2I of 500, 400, 300 ml/min/m^2 (time <300, <400, <500).	6
**Supplemental Figure S3**	The receiver operating characteristic curves to predict posttransplant acute kidney injury of the area under the threshold of oxygen delivery index (DO_2I) of 300 ml/min/m^2 (AUC<300), cumulative time below the threshold of DO_2I of 300 ml/min/m^2 (time<300 minutes), mean, nadir, and standard deviation (SD) of intraoperative DO_2I.	7
**Supplemental Figure S4**	Histograms of the distribution of standardized differences and covariate balance plot before and after matching.	8

Supplemental Table S1. Patient characteristics and perioperative parameters of the patients with high and low mean oxygen delivery index (mean DO_2I) after propensity score matching.

Characteristic	Mean DO_2I ≥ 500 ml/min/m^2	Mean DO_2I < 500 ml/min/m^2	P-value
Sample size	192 (50.0)	192 (50.0)	
Demographic data			
Age, years	54 (49 - 61)	55 (51 - 62)	0.611
Female, n	59 (30.7)	66 (34.4)	0.446
Body-mass index, kg/m^2	23.2 (21.2 - 25.3)	23.0 (21.0 - 25.5)	0.312
Etiology of liver disease			
Alcoholic liver cirrhosis, n	21 (10.9)	24 (12.5)	0.634
Hepatocellular carcinoma, n	76 (39.6)	75 (39.1)	0.917
Baseline medical status			
Hypertension, n	29 (15.1)	33 (17.2)	0.579
Diabetes mellitus, n	31 (16.1)	33 (17.2)	0.784
Preoperative hemoglobin, g/dL	10.7 (9.3 - 12.1)	10.3 (9.0 - 12.1)	0.785
Preoperative serum albumin level, mg/dL	2.9 (2.5 - 3.3)	3.0 (2.6 - 3.4)	0.620
Model for end-stage liver disease score	15 (11 - 22)	15 (11 - 23)	0.835
Child class, n (A/ B/ C)	51 (26.6)/ 81 (42.2)/ 60 (31.3)	50 (26.0)/ 82 (42.7)/ 60 (31.3)	0.992
Preoperative beta-blocker, n	14 (7.3)	15 (7.8)	0.847
Preoperative diuretics, n	10 (5.2)	9 (4.7)	0.814
Previous abdominal surgery, n	6 (3.1)	7 (3.6)	0.999
Donor/ graft factors			
Living/ deceased donor, n	116 (60.4)/ 76 (39.6)	115 (59.9)/ 77 (40.1)	0.917
Estimated GRWR	1.23 (1.06-1.47)	1.20 (1.08-1.46)	0.884
Operation and anesthesia details			
Operation time, hour	6.7 (5.9 - 7.8)	6.4 (5.3 - 7.9)	0.889
Cold ischemic time, min	105 (72 - 240)	104 (72 - 240)	0.906
Warm ischemic time, min	30 (27 - 32)	30 (26 - 34)	0.992
Intraoperative dose of epinephrine bolus, mcg	5 (0 - 30)	10 (0 - 40)	0.618
pRBC transfusion, units	7 (3-14)	7 (2-14)	0.869

The values are expressed as the median [interquartile range] or number (%). GRWR = graft recipient body-weight ratio, pRBC = packed red blood cells.

Supplemental Table S2. The incidence of acute kidney injury (AKI) according to the groups of high and low model for end-stage liver disease (MELD) score or Child class and cumulative time below intraoperative oxygen delivery index < 300 ml/min/m^2 (time <300).

The incidence of AKI with high time <300 and high MELD score or Child class C was significantly higher than that of AKI with high time <300 and low MELD score or Child class A or B (*P* <0.001 after Bonferroni correction for multiple testing.

Characteristic	Time <300<br>≤ 30 minutes<br>(n = 429)	Time <300<br>> 30 minutes<br>(n = 247)	*P*-value
Low MELD score (≤ 20, n = 483)			
AKI, n	76 (23.6)	100 (56.8)	<0.001
No AKI, n	246 (76.4)	61 (37.9)	
High MELD score (> 20, n = 193)			
AKI, n	32 (29.9)	67 (77.9)	<0.001
No AKI, n	75 (70.1)	19 (22.1)	
Child class A (n = 207)			
AKI, n	20 (13.6)	35 (58.3)	<0.001
No AKI	127 (86.4)	25 (41.7)	
Child class B or C (n = 469)			
AKI, n	88 (31.2)	132 (70.6)	<0.001
No AKI, n	194 (68.8)	55 (29.4)	

The values are expressed as or number (%).

Supplemental Figure S1. Comparison of serum hemoglobin concentration (upper), arterial oxygen partial pressure (middle) and arterial oxygen saturation (lower) during liver transplantation between the patients with and without acute kidney injury.

The time points compared were as follows; anesthesia induction (T1), 1 hour after anesthesia induction (T2), 30 min (T3) and 1 hour (T4) after the beginning of the anhepatic phase, 5 min before (T5) and after (T6) graft reperfusion, 20 min after reperfusion (T7), 40 min after reperfusion (T8), 5 min after the completion of biliary reconstruction(T9), and at the end of surgery (T10). There was no significant difference in any time points between groups.

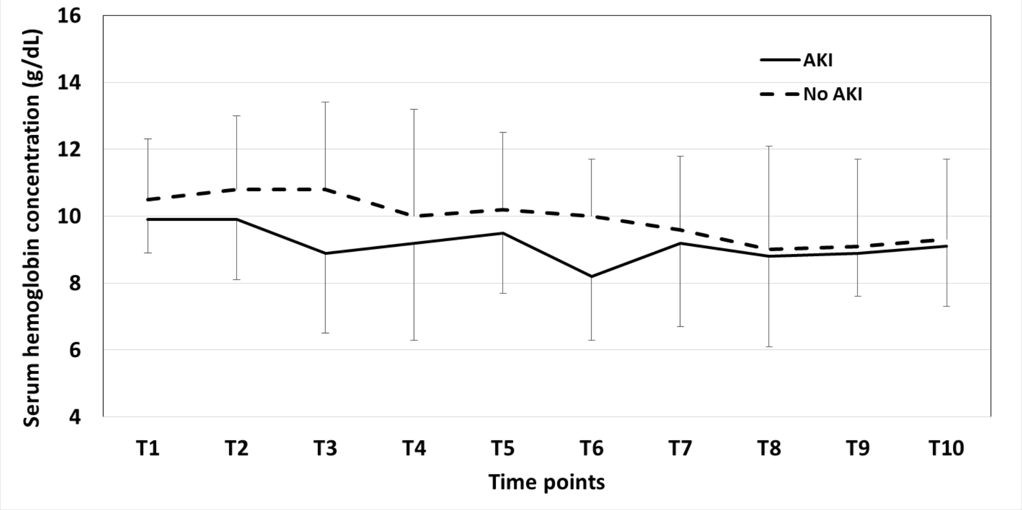

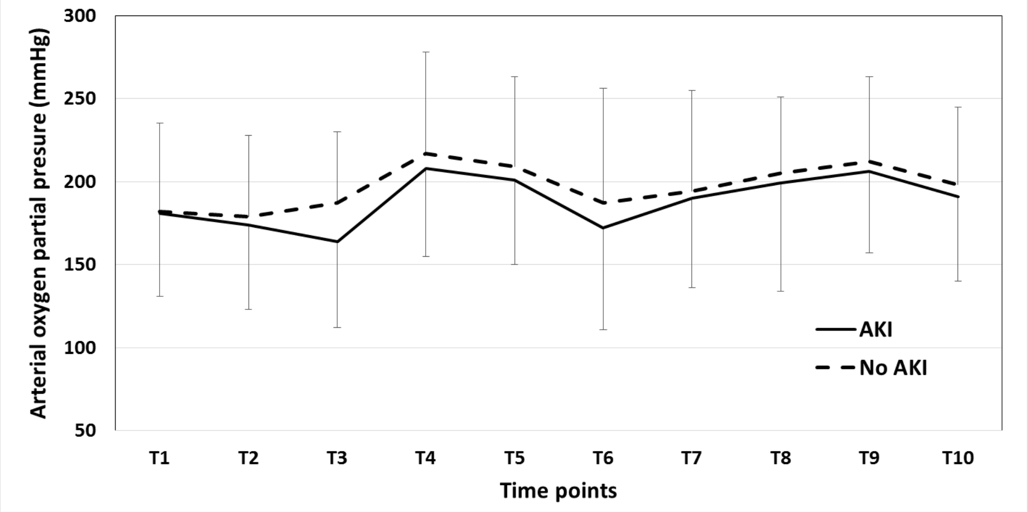

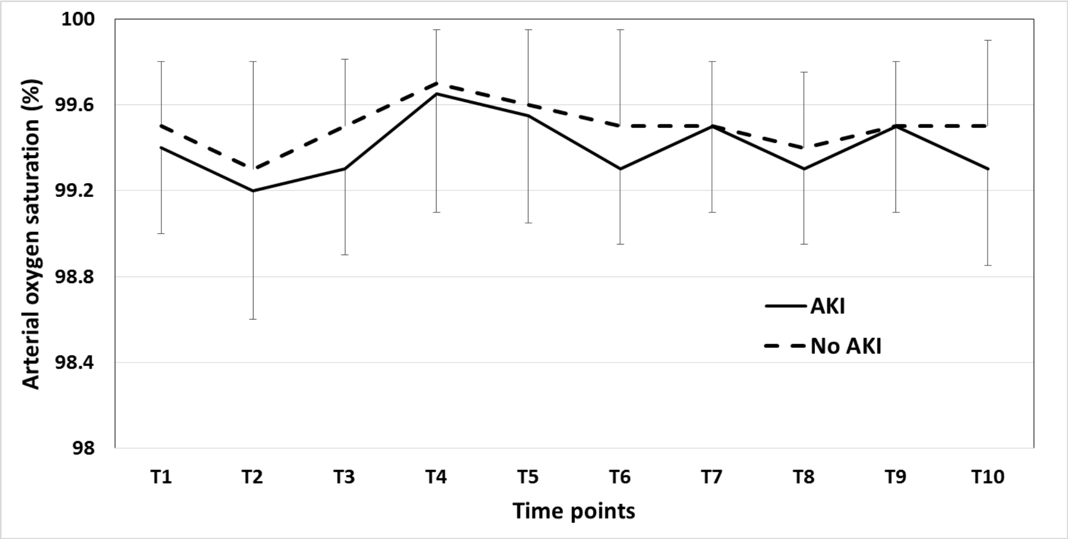
100
99.6
99.2
98.8
98.4
98
Arterial oxygen saturation (%)
AKI
No AKI
T1
T2
T3
T4
T5
T6
T7
T8
T9
T10
Time points

Supplemental Figure S2. The receiver operating characteristic curves to predict posttransplant acute kidney injury of the area under the threshold of oxygen delivery index (DO_2I) of 500, 400, 300 ml/min/m^2 (AUC <300, <400, <500), and cumulative time (minutes) below the threshold of DO_2I of 500, 400, 300 ml/min/m^2 (time <300, <400, <500).

The areas under the receiver operating characteristic curves (AUROC) were 0.72 (95% confidence interval [CI] 0.68 – 0.75), 0.68 (95% CI 0.65 – 0.72), 0.58 (95% CI 0.54 – 0.61) for AUC <300, AUC <400, AUC <500, respectively, and 0.72 (95% CI 0.68 – 0.75), 0.69 (95% CI 0.65 – 0.72), 0.61 (95% CI 0.57 – 0.65) for time <300, time <400, time <500, respectively. AUROC of AUC <300 was significantly larger than that of AUC <400 or AUC <500. AUROC of time <300 was significantly larger than that of time <400 or AUC <500.

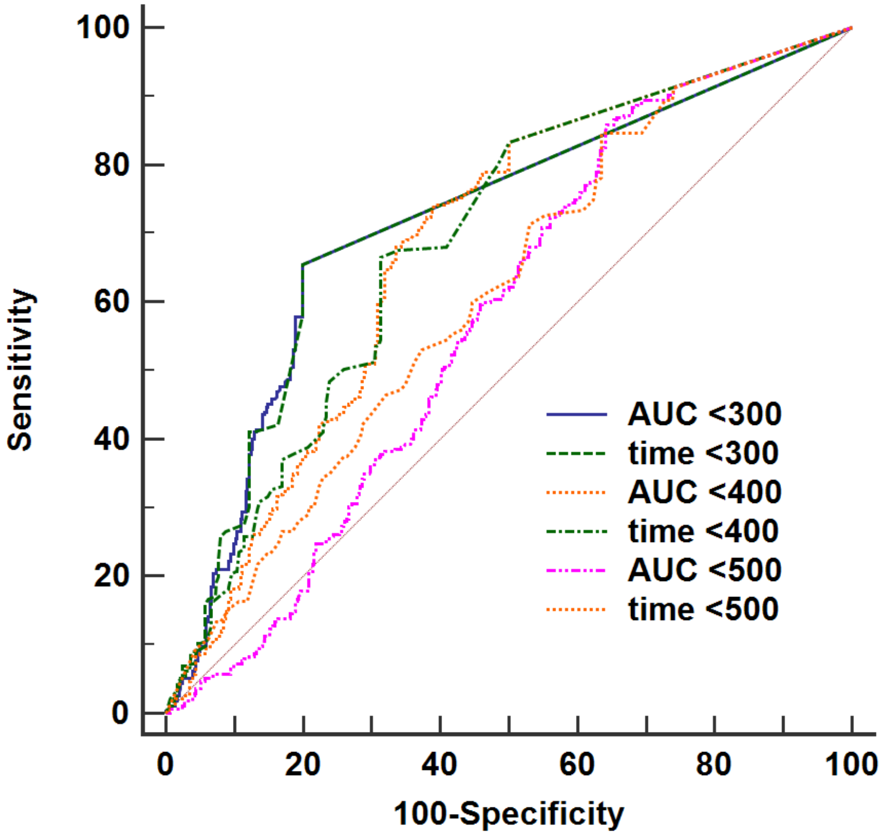

Supplemental Figure S3. The receiver operating characteristic curves to predict posttransplant acute kidney injury of the area under the threshold of oxygen delivery index (DO_2I) of 300 ml/min/m^2 (AUC<300), cumulative time below the threshold of DO_2I of 300 ml/min/m^2 (time<300 minutes), mean, nadir, and standard deviation (SD) of intraoperative DO_2I.

The area under the receiver operating characteristic curves (AUROC) were 0.72 (95% confidence interval [CI] 0.68–0.75), 0.72 (95% CI 0.68–0.75), 0.65 (95% CI 0.61–0.69), 0.67 (95% CI 0.64–0.71), and 0.67 (95% CI 0.63–0.71) for AUC<300, time<300, mean DO_2I, nadir DO_2I, and SD of DO_2I. The AUROC of AUC<300 and time<300 were significantly larger than that of mean or nadir DO_2I.

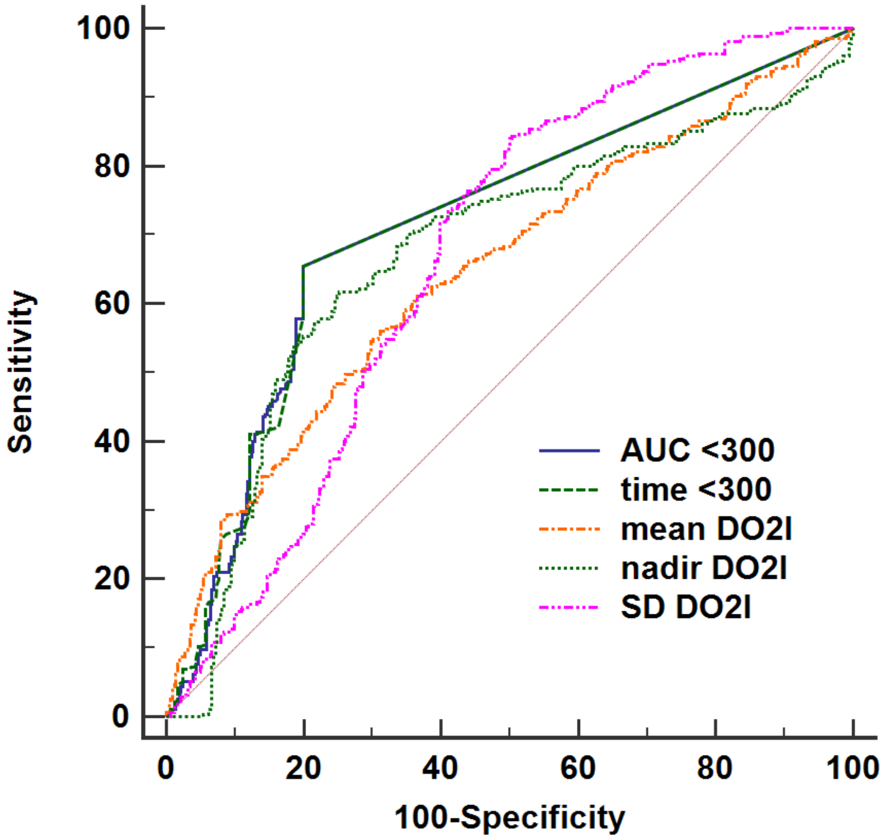

Supplemental Figure S4. Histograms of the distribution of standardized differences and covariate balance plot before and after matching.

LDLT = living-donor liver transplantation; DDLT = deceased-donor liver transplantation; CIT = cold ischemic time; WIT = warm ischemic time; GRWR = graft recipient body-weight ratio, RBC_tf = red blood cells transfusion; premedi_Hb = preoperative hemoglobin; premedi_Alb = preoperative albumin; HTN = hypertension; DM = diabetes mellitus; HCC = hepatocellular carcinoma.

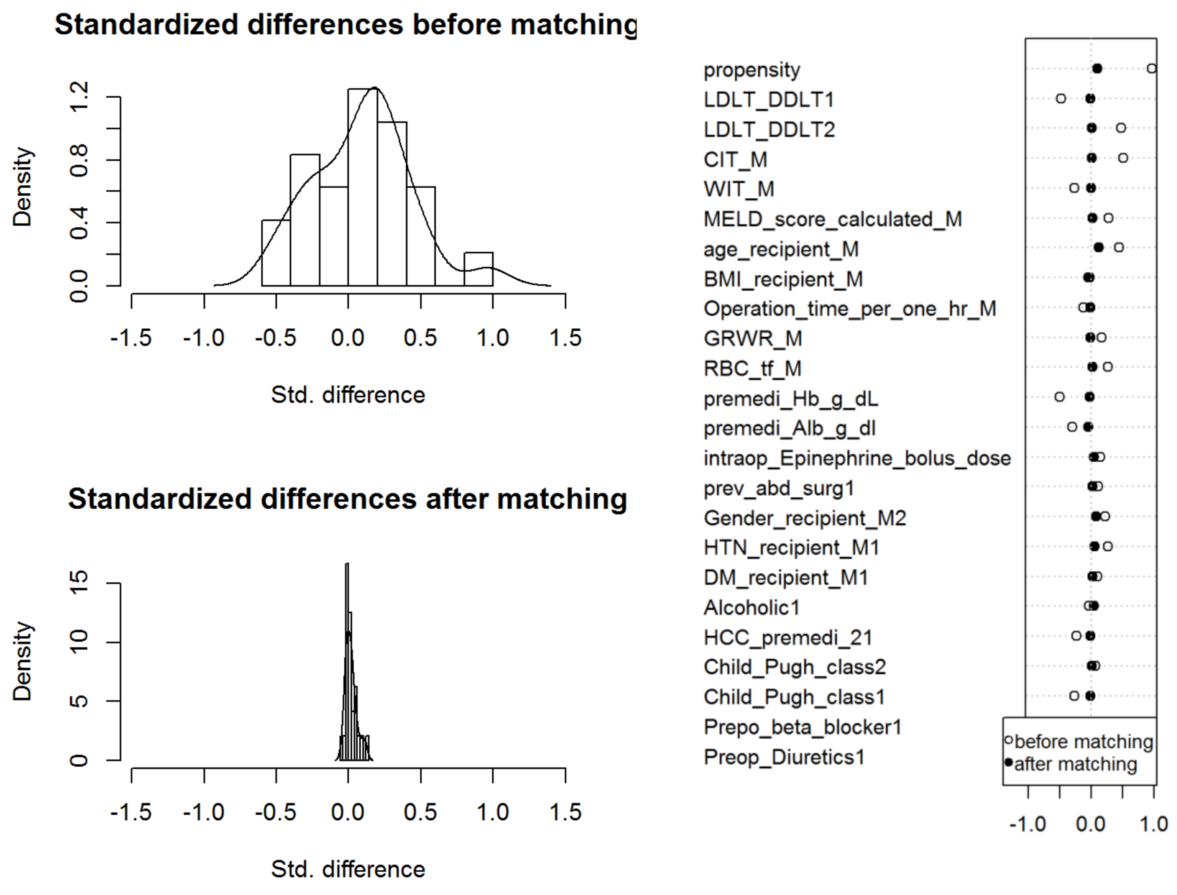

Supplement: Supplementary file 1 [file jcm-09-00564-s001.pdf]
